# Characterization of extracellular vesicles from *Lactiplantibacillus plantarum*

**DOI:** 10.1038/s41598-022-17629-7

**Published:** 2022-08-08

**Authors:** Atsushi Kurata, Shogo Kiyohara, Tomoya Imai, Shino Yamasaki-Yashiki, Nobuhiro Zaima, Tatsuya Moriyama, Noriaki Kishimoto, Koichi Uegaki

**Affiliations:** 1 Department of Applied Biological Chemistry, Faculty of Agriculture, Kindai University, 204-3327 Nakamachi, Nara, Nara 631-8505 Japan; 2 Research Institute for Sustainable Humanosphere, Kyoto University, Uji, Kyoto 611-0011 Japan; 3 Department of Life Science and Biotechnology, Faculty of Chemistry, Materials and Bioengineering, Kansai University, 3-3-35 Yamate-cho, Suita, Osaka 564-8680 Japan; 4 Agricultural Technology and Innovation Research Institute, Kindai University, 204-3327 Nakamachi, Nara, Nara 631-8505 Japan

**Keywords:** Bacteria, Bacterial host response

## Abstract

We investigated the characteristics and functionalities of extracellular vesicles (EVs) from *Lactiplantibacillus plantarum* (previously *Lactobacillus plantarum*) towards host immune cells. *L*. *plantarum* produces EVs that have a cytoplasmic membrane and contain cytoplasmic metabolites, membrane and cytoplasmic proteins, and small RNAs, but not bacterial cell wall components, namely, lipoteichoic acid and peptidoglycan. In the presence of *L*. *plantarum* EVs, Raw264 cells inducibly produced the pro-inflammatory cytokines IL-1β and IL-6, the anti-inflammatory cytokine IL-10, and IF-γ and IL-12, which are involved in the differentiation of naive T-helper cells into T-helper type 1 cells. IgA was produced by PP cells following the addition of EVs. Therefore, *L*. *plantarum* EVs activated innate and acquired immune responses. *L*. *plantarum* EVs are recognized by Toll-like receptor 2 (TLR2), which activates NF-κB, but not by other TLRs or NOD-like receptors. *N*-acylated peptides from lipoprotein19180 (Lp19180) in *L*. *plantarum* EVs were identified as novel TLR2 ligands. Therefore, *L*. *plantarum* induces an immunostimulation though the TLR2 recognition of the *N*-acylated amino acid moiety of Lp19180 in EVs. Additionally, we detected a large amount of EVs in the rat gastrointestinal tract for the first time, suggesting that EVs released by probiotics function as a modulator of intestinal immunity.

## Introduction

The animal gastrointestinal tract is inhabited by a complex community of bacteria called the gut microbiota. These bacteria stimulate the intestinal immune system, which contributes to the maintenance of host gastrointestinal homeostasis^1,2^. Some symbiotic gut bacteria are transported into the intestinal lamina propria by the microfold (M) cells of Peyer’s patches (PP). Transported bacteria subsequently induce immune responses in PP cells containing macrophages, B cells, T cells, and other immune cells. Innate immune cells including macrophages recognize various bacterial components by pattern recognition receptors (PRRs), such as Toll-like receptors (TLRs) and NOD-like receptors (NLRs), to produce a number of cytokines via the activation of the transcription factor NF-κB^3^. Cytokines and other activators then stimulate acquired immune cells, including B cells, to produce immunoglobulin (Ig) A^4,5^. A detailed understanding of the products of bacterial cells that affect host immune cells is of importance.

The symbiotic intestinal bacteria that are beneficial to living organisms are called probiotics. Lactobacilli have been attracting increasing attention as typical probiotic Gram-positive bacteria. Several lactobacilli produce extracellular vesicles (EVs) with a spherical structure that range in size between 20 and 200 nm in culture medium^6,7^. EVs are presumed to carry metabolic intermediates, proteins, and RNAs as cargo and stimulate host immune cells. The EVs of *Latilactobacillus sakei* (previously *Lactobacillus sakei*) have been shown to induce the production of IgA from PP cells^8,9^. The pro-inflammatory cytokine IL-6, derived from dendritic cells, enhances IgA production by *L*. *sakei* EVs. The EVs of *Lacticaseibacillus rhamnosus* (previously *Lactobacillus rhamnosus*) GG and *Lactobacillus reuteri* DSM 17938 were found to weaken the pro-inflammatory cytokine responses of T cells and NK cells^10^. Although the function of EVs in the host immune system is important, the molecules in EVs that are responsible for immunostimulation remain unknown. To date, the biochemical components of EVs have been largely unidentified. Therefore, further studies are warranted to identify the biochemical components of EVs as well as the molecules responsible for triggering immunostimulatory effects.

We focused on lipoprotein (Lp) as a functional molecule of EVs. The important function of Lp in Gram-positive bacteria has only emerged in recent years. Lp is anchored to the bacterial cytoplasmic membrane via a di- or tri-acylglycerol moiety linked to the *N*-terminal cysteine of Lp^11^. The polypeptide portion of Lp protrudes outside the Gram-positive bacterial cell surface and is responsible for characteristic functions, such as the uptake of nutrients. Some pro-inflammatory cytokines are inducibly produced by host immune cells following the addition of an acylated *N*-terminal peptide of Lp from *Staphylococcus aureus*^12^. However, the effects of Lp from lactobacilli on host immune cells remain unclear. Lp in EVs released from lactobacilli have not yet been investigated.

**Figure 1:**
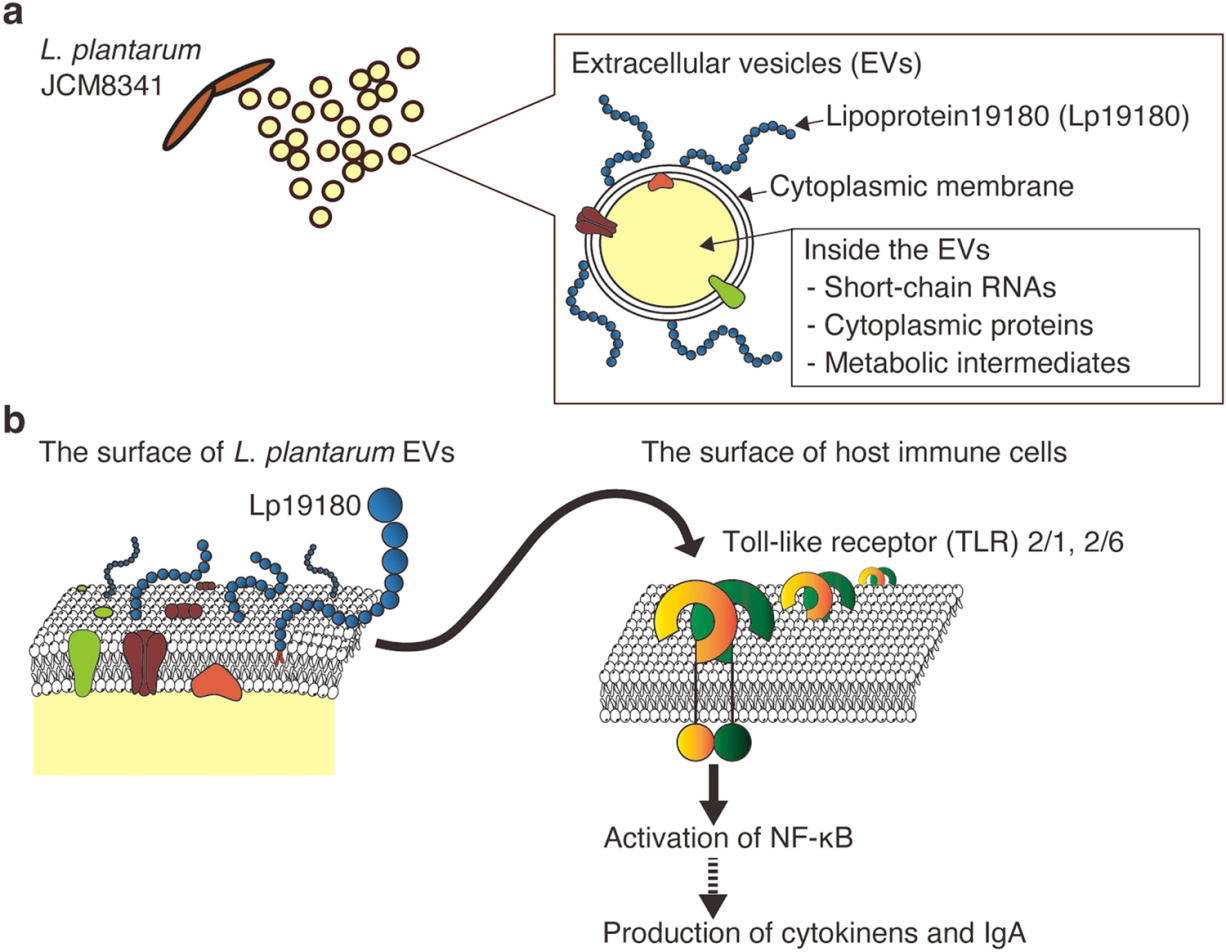
Properties of EVs produced by *L*. *plantarum* and their effects on host immune cells. (**a**) Biochemical components of *L*. *plantarum* EVs. (**b**) Schematic sketch of NF-κB activation by Lp19180 via TLR2 and the subsequent stimulation of innate and adaptive immunities.

We newly identified *Lactiplantibacillus plantarum* (previously *Lactobacillus plantarum*) JCM8341 as a producer of EVs. Using EVs produced by *L*. *plantarum*, the actual number and size of EVs with a bacterial cytoplasmic membrane were quantified with a fluorescent dye that specifically stains the cytoplasmic membrane. To identify the biochemical components of *L*. *plantarum* EVs, metabolomic, proteomic, and RNA-seq analyses were performed. To date, the stimulation of innate immunity by whole cells of *L*. *plantarum* JCM8341 has been reported^13^. In the present study, we showed that EVs from *L*. *plantarum* JCM8341 activated NF-κB via TLR2 recognition and induced the production of pro- and anti-inflammatory cytokines from Raw264 cells and IgA from PP cells. Therefore, *L*. *plantarum* EVs are involved in the activation of innate and acquired immune responses. We also detected a novel Lp (named Lp19180) in *L*. *plantarum* EVs. The acylated *N*-terminal peptides derived from Lp19180 were recognized by TLR2 and activated NF-κB. In summary, we herein report the physicochemical, biochemical, and functional characteristics of *L*. *plantarum* EVs (Fig. 1). Furthermore, to elucidate the effects of EVs against gut immunity in vivo, we measured the numbers and sizes of EVs in the rat gastrointestinal tract. The present results will provide insights into the effects of lactobacillus EVs on host immunity at the molecular level.

## Results

### Physicochemical characterization of *L*. *plantarum* EVs

**Figure 2:**
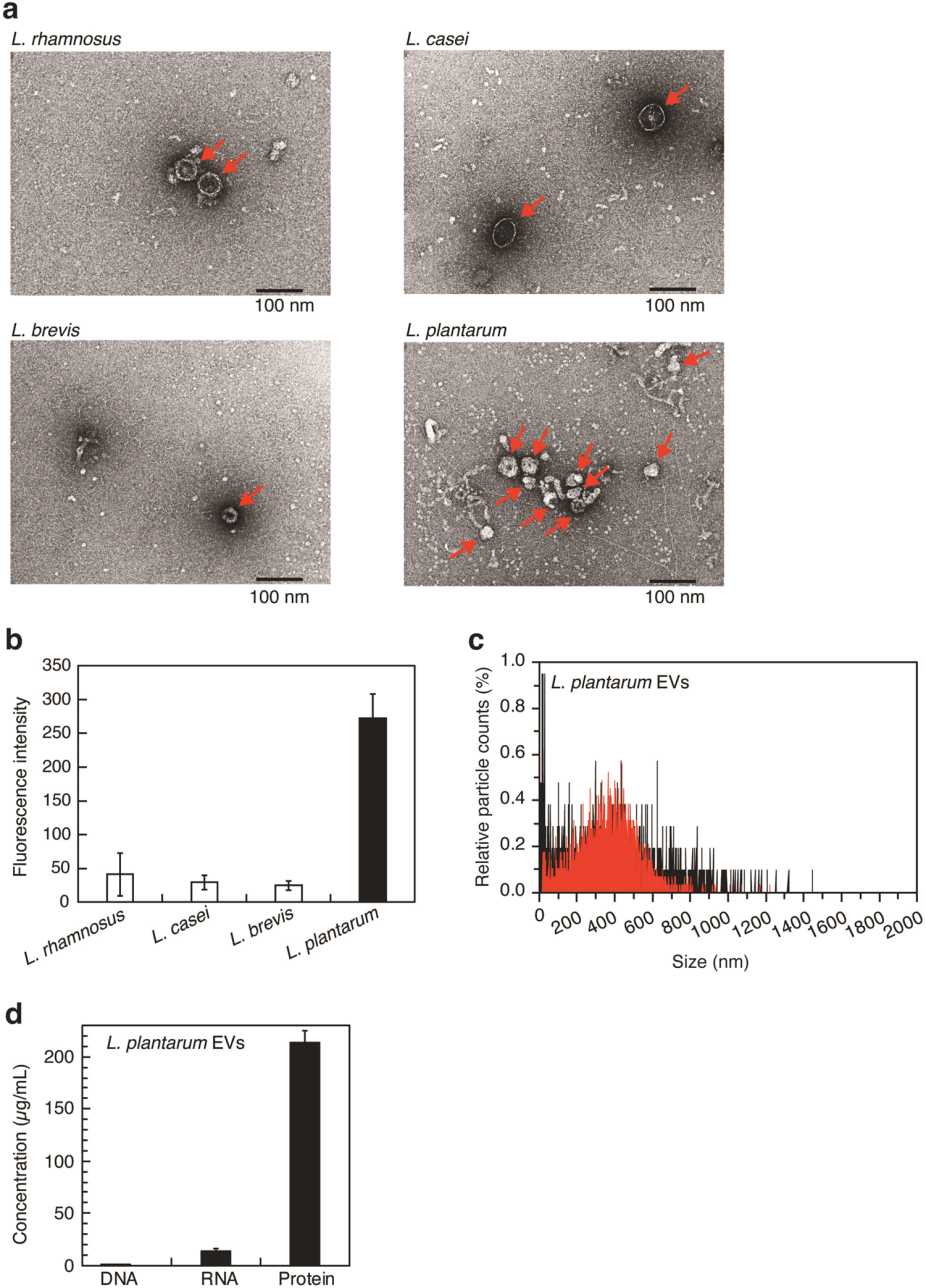
Characterization of EVs produced by *L*. *plantarum.* (**a**) TEM images of lactobacillus EVs. Red arrows indicate EVs. Scale bar, 100 nm. (**b**) EVs production of lactobacilli. Means ± SD, n = 3. (**c**) Size distributions of EVs (black: all particles in the *L*. *plantarum* EVs fraction, red: EVs labeled with FM4-64). The total numbers of particles in *L*. *plantarum* EVs and those of particles labeled by FM4-64 in *L*. *plantarum* EVs are represented as 100%. (**d**) DNA, RNA, and protein in *L*. *plantarum* EVs. Means ± SD, n = 3.

We attempted to detect the spherical structures (20–200 nm) of EVs in the culture supernatant of lactobacilli (*Lacticaseibacillus rhamnosus* JCM2772, *Lacticaseibacillus casei* JCM 1134, *Levilactobacillus brevis* JCM1059^T^, and *L. plantarum* JCM8341). Using transmission electron microscopy (TEM), we investigated whether these spherical structures were present in the ultracentrifugation residue obtained from the bacterial culture supernatant by differential centrifugation. As shown in Fig. 2a, we confirmed the presence of structures with a diameter of approximately 50 nm (red arrow) in the ultracentrifugation residues of four lactobacilli strains. As shown in Fig. 2b, EVs from four lactobacilli strains were quantified using the lipophilic fluorescent dye, (*N*-(3-triethylammoniumpropyl)-4-(6-(4-(diethylamino) phenyl) hexatrienyl) pyridinium dibromide (FM4-64). FM4-64 is virtually non-fluorescent in aqueous solution, but becomes intensely fluorescent when the dye is incorporated into cytoplasmic membranes^14^. Of the four lactobacilli, *L*. *plantarum* produced large amounts of EVs. Fluorescence nanoparticle tracking analysis with FM4-64 was then performed to evaluate the actual numbers and sizes of particles with a cytoplasmic membrane in the culture supernatant of *L*. *plantarum* (Fig. 2c). We detected particles ranging in size between 10 and 1500 nm (Fig. 2c, black lines). Membrane particles (50–800 nm) labeled with FM4-64 were observed (Fig. 2c, red lines) and many membrane particles of approximately 400 nm were present. The results of TEM observations shown in Fig. 2a revealed that *L*. *plantarum* produced vesicle-like structures (approximately 50 nm). The zeta potential of *L*. *plantarum* EVs was −73.32 ± 22.33 mV, indicating that EVs are weakly charged and potentially form multimer structures in aqueous solution. These results suggest that *L*. *plantarum* EVs exist as monomer (50 nm) and multimer (~ 800 nm) structures in aqueous solution. In summary, *L*. *plantarum* produced at least 4.5 × 10^10^ particles/ml of EVs consisting of a cytoplasmic membrane with a size range of 50–800 nm in culture medium. *Lactobacillus acidophilus* ATCC 53544, *L*. *casei* ATCC 393, and *L*. *reuteri* ATCC 23272 were previously reported to produce between 3 × 10^9^ and 1 × 10^10^ particles/ml of EVs in each culture medium^6^. The concentration of *L*. *plantarum* EVs detected in culture medium was consistent with those of EVs from these lactobacilli in culture media. The results of TEM observations also showed that the sizes of EVs produced by *L*. *plantarum* WCFS1, KCTC 11401BP, and BGAN8 were 30–200, 20–100, and 20–140 nm, respectively^15–17^. As shown in Fig. 2a, *L*. *plantarum* EVs had similar structures. Additionally, protein and RNA were present in *L*. *plantarum* EVs, whereas DNA was rarely detected (Fig. 2d). This result is similar to that reported for *L*. *reuteri*^18^. To summarize, some lactobacilli release EVs into culture supernatants. *L*. *plantarum* EVs appear to have a cytoplasmic membrane and mainly contain bacterial proteins.

### Comprehensive analysis of constituents of *L*. *plantarum* EVs

To the best of our knowledge, few studies to date have conducted a biochemical analysis of the constituents of EVs. Therefore, we attempted to comprehensively identify the constituent molecules of *L*. *plantarum* EVs using a metabolomic analysis with LC–MS. The results obtained revealed the presence of 1519 compounds in *L*. *plantarum* EVs (Supplementary Table S1). Some of the components in *L*. *plantarum* EVs are shown in Table 1. Eicosatetraenoyl-glycerophosphate and various fatty acids, which constitute the bacterial cytoplasmic membrane, were detected in EVs. The following fatty acids have been detected in whole cells of *L*. *plantarum*; C12:0, C12:1, C14:0, C14:1, C16:0, C16:1, C18:0, C18:1, and C20:1^19,20^. As shown in Table 1, oxododecanoic acid (C12:0), oxotetradecanoic acid (C14:0), palmitoleic acid (C16:1), and oleic acid (C18:1) were identified in *L*. *plantarum* EVs. These results support the assumption that EVs are composed of the cytoplasmic membrane of *L*. *plantarum*, as shown in Fig. 2c. Additionally, we detected NAD^+^, amino acids, and nucleic acids in *L*. *plantarum* EVs. The EVs of Gram-positive bacteria have been suggested to bud from the cytoplasmic membrane^21^, and cytoplasmic metabolites, namely, NAD^+^, amino acids, and nucleic acid, may be contained as cargo inside *L*. *plantarum* EVs. The presence of lipoteichoic acid (LTA) and peptidoglycan (PGN) in the bacterial cell wall has been reported in the EVs of *L*. *rhamnosus* JB-1 and *S*. *aureus* strains 6571, respectively^22,23^. On the other hand, in *L*. *plantarum* EVs, we did not detect teichoic acid, glycerol phosphate, or ribitol phosphate derived from LTA, or γ-D-glutamyl-meso-diaminopimelic acid (iE-DAP), muramyldipeptide (MDP), *N*-acetylglucosamine, or *N*-acetylmuramic acid derived from PGN. In summary, *L*. *plantarum* EVs are vesicles that have a bacterial cytoplasmic membrane and contain a number of cytoplasmic metabolites. Bacterial cell wall components, namely, LTA and PGM, are not detected in *L*. *plantarum* EVs.

**Table 1:** Constituents in *L*. *plantarum* EVs.

		Peak no.	R.T. (min)	Ionization	*m*/*z* (detected)	Exact mass
**Cytoplasmic membrane**						
Phospholipids	Eicosatetraenoyl-glycerophosphate	569	15.10	[M + H]^+^	459.2506	458.2433
Fatty acids	Propionyl-CoA	851	16.37	[M + H]^+^	824.1474	823.1414
	Oxododecanoic acid	1185	23.25	[M + H]^+^	215.1643	214.1569
	Oxotetradecanoic acid	1291	25.24	[M + H]^+^	243.1955	242.1882
	Palmitoleic acid	1362	26.47	[M + H]^+^	255.2319	254.2246
	Hydroxyarachidonic acid	1420	27.21	[M + H]^+^	321.2424	320.2351
	Oleic acid	1485	29.01	[M + H]^+^	283.2632	282.2559
**Cytoplasm**						
Coenzyme	NAD^+^	61	3.47	[M + 2H]^2+^	332.5618	663.1091
Amino acids	Tryptophan	223	12.06	[M + H]^+^	205.0973	204.0899
	Tyrosine	1000	19.82	[M + H]^+^	182.0814	181.0739
	Leucine/isoleucine	1314	25.82	[M + H]^+^	132.1021	131.0946
Nucleic acids	Guanine	44	3.45	[M + H]^+^	152.0569	151.0494
	Adenine	45	3.45	[M + H]^+^	136.0619	135.0545
	Ribothymidine	91	5.28	[M + H]^+^	259.0925	258.0852
	Adenosine	98	6.59	[M + H]^+^	268.1042	267.0968

*R.T*. retention time.

### Proteomic analysis of *L*. *plantarum* EVs

**Figure 3:**
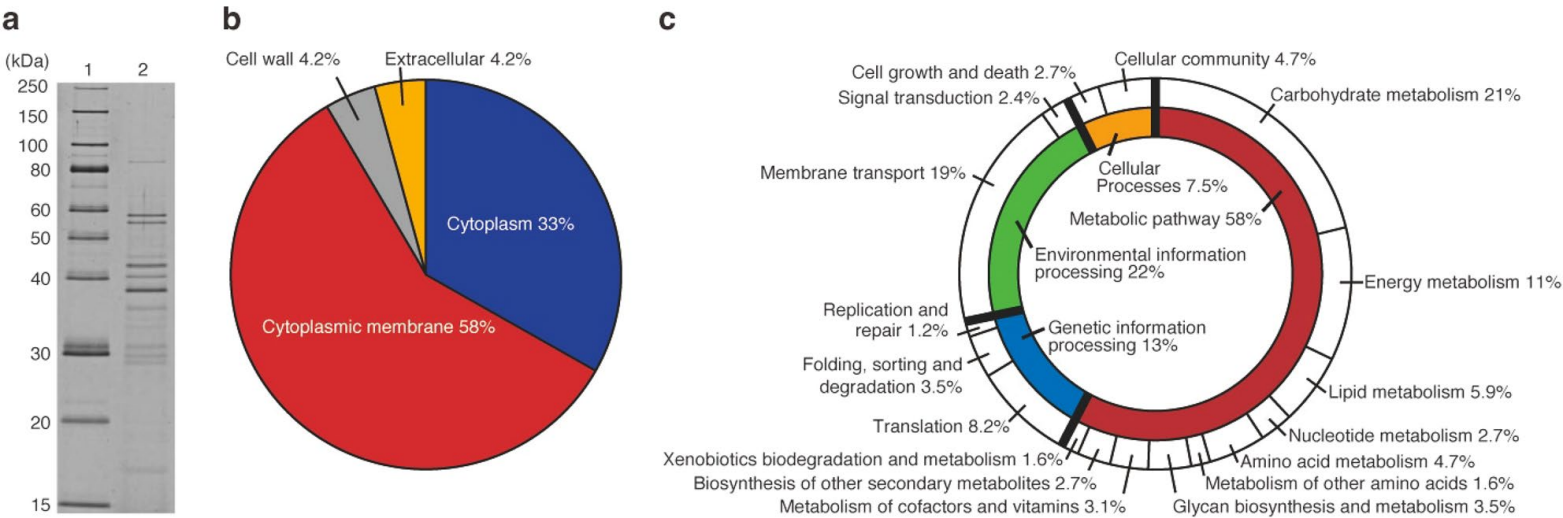
Proteins in *L*. *plantarum* EVs. (**a**) SDS-PAGE profile of *L*. *plantarum* EVs. Lane 1: Marker, Lane 2: *L*. *plantarum* EVs (0.3 µg). Proteins were separated by SDS-PAGE using 12.5% gel (Gellex International, Tokyo, Japan) with constant voltage at 300 V and then detected using SYPRO Ruby staining (Lonza, Rockland, ME). The cropped gel was displayed, and original gel was indicated in Supplementary Fig. 3a_original_gel file. (**b**) Subcellular localization of proteins in *L*. *plantarum* EVs. (**c**) Biological processes involving proteins in *L*. *plantarum* EVs.

We attempted to identify the proteins detected in *L*. *plantarum* EVs (Fig. 2d). *L*. *plantarum* EVs collected from the supernatants of three independent cultures were analyzed by SDS-PAGE. The protein profiles of *L*. *plantarum* EVs were highly reproducible among the three replicates. A representative SDS-PAGE profile is shown in Fig. 3a. As a result of sequencing of the genomic DNA of *L*. *plantarum* (Genbank Accession No. BPFY01000000), we identified 3031 types of protein-coding genes in genomic DNA. Using the amino acid sequences of proteins in genomic DNA as reference data, 411 types of proteins in *L*. *plantarum* EVs were comprehensively identified by LC–MS/MS. The proteins identified in *L*. *plantarum* EVs and their predicted subcellular localization are shown in Supplementary Table S2. Predictions of the subcellular localization of EV proteins were performed using PSORTb 3.0.2 (https://www.psort.org/psortb/). Following the exclusion of 58 types of hypothetical proteins from the 411 types in *L*. *plantarum* EVs, we predicted the subcellular localization of 283 out of 353 types of proteins in EVs (Fig. 3b). Many proteins in *L*. *plantarum* EVs were predicted to localize in the bacterial cytoplasmic membrane and cytoplasm of *L*. *plantarum* cells, and a small number to the cell wall or extracellular environment.

As shown in Fig. 3c, the biological functions of proteins in *L*. *plantarum* EVs were cataloged using to KEGG Mapper (https://www.genome.jp/kegg/mapper.html). Among the 353 types of EV proteins described above, 255 were evaluated for biological functions (Supplementary Table S3), with 54 being involved in carbohydrate metabolism (21%), followed by 49 in membrane transport (19%) (Fig. 3c). As shown in Fig. 2c and Table 1, *L*. *plantarum* EVs were composed of a cytoplasmic membrane. Additionally, cytoplasmic metabolites and proteins appeared to be present inside the spherical structures of EVs. Therefore, we considered a large number of membrane and cytoplasmic proteins to be present in *L*. *plantarum* EVs. Similar to *L*. *plantarum* EVs, membrane and cytoplasmic proteins involved in diverse metabolic pathways have been detected in *L*. *plantarum* BGAN8 EVs and *L*. *reuteri* BBC3 EVs^15,18^.

We identified lysozyme (LOCUS_18750) and the cell wall amidase lytH (LOCUS_29150) in *L*. *plantarum* EVs, as shown in Supplementary Table S2. Since these enzymes are responsible for bacterial cell wall degradation^24^, the LTA and PGN components of the bacterial cell wall may not have been detectable in *L*. *plantarum* EVs. In addition, EVs are released from Gram-positive bacterial cells when the cytoplasmic membrane protrudes outside due to turgor pressure when the bacterial cell wall is locally thinned by these enzymes^21^. In the case of *L*. *plantarum*, EVs may be released from bacterial cells by the same mechanism.

### Analysis of small RNAs in *L*. *plantarum* EVs

**Figure 4:**
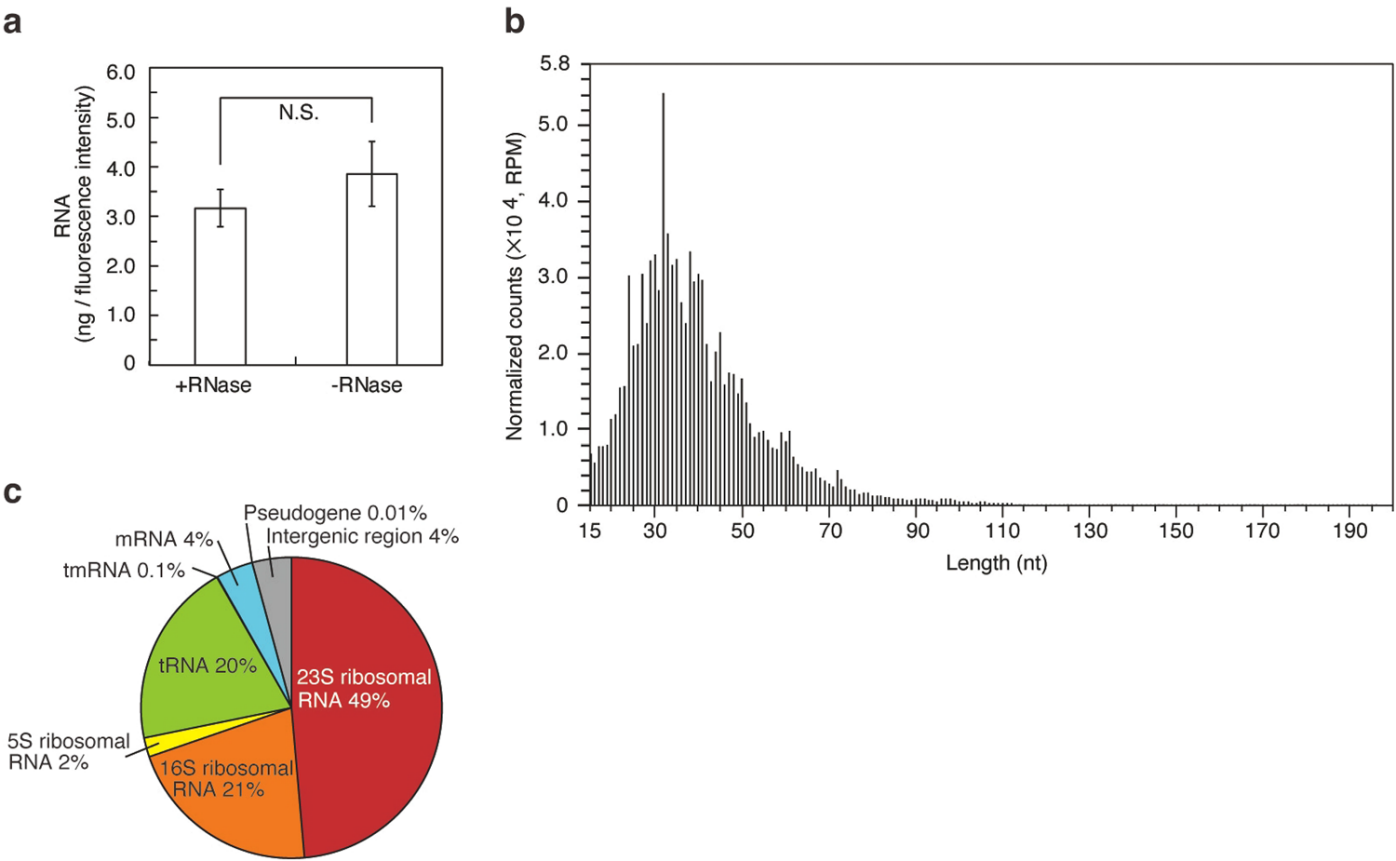
Small RNAs in *L*. *plantarum* EVs. (**a**) The amount of RNA per fluorescence intensity of EVs. The amount of RNA after the RNase treatment was compared with that without the treatment. Means ± SD, n = 3. Two-tailed unpaired Student’s t-test, *N.S.* not significant. (**b**) Strand length distribution of RNAs. (**c**) Classification of small RNAs in *L*. *plantarum* EVs.

We detected RNAs (Fig. 2d) and nucleic acids (Table 1) in *L*. *plantarum* EVs and attempted to analyze the former. RNAs in *L*. *plantarum* EVs from the supernatants of three independent cultures were quantified, and representative EVs containing RNA were used in analyses. We initially investigated whether RNA was degraded by an RNase treatment. As shown in Fig. 4a, the amount of RNA in EVs treated with RNase was similar to that in EVs not treated with RNase (*p* = 0.18). This result suggested that RNA was encapsulated in *L*. *plantarum* EVs, which protected it from degradation by RNase, similar to *L*. *reuteri* BBC3 EVs^18^. Small RNA-seq was then performed to comprehensively analyze the nucleotide sequences and strand lengths of RNA in *L*. *plantarum* EVs (Fig. 4b). We elucidated the nucleotide sequences and strand lengths of 406,312 reads of RNAs. Small RNA-seq data are shown in Supplementary Table S4. As shown in Fig. 4b, the length of RNA encapsulated in EVs ranged between 15 and 196 nt. Mean and mode chain lengths were 42 and 32 nt, respectively. RNAs in *L*. *plantarum* EVs were mapped using the *L*. *plantarum* genomic DNA sequence (Genbank Accession No. BPFY01000000). The results obtained revealed that 72% of small RNAs in *L*. *plantarum* EVs were derived from 5S, 16S, and 23S rRNA of *L*. *plantarum* (Fig. 4c). We detected 20 types of large and small subunits of ribosomal proteins in *L*. *plantarum* EVs (Supplementary Table S3). The mechanisms underlying RNA intake in bacterial EVs have not yet been elucidated in detail; however, proteins appear to be synthesized close to the membrane site, resulting in the capture of rRNA, tRNA, and mRNA fragments along with ribosomal proteins in bacterial EVs^21^. Therefore, *L*. *plantarum* EVs are assumed to contain small RNAs and ribosomal proteins.

### Effects of *L*. *plantarum* EVs against immune cells

**Figure 5:**
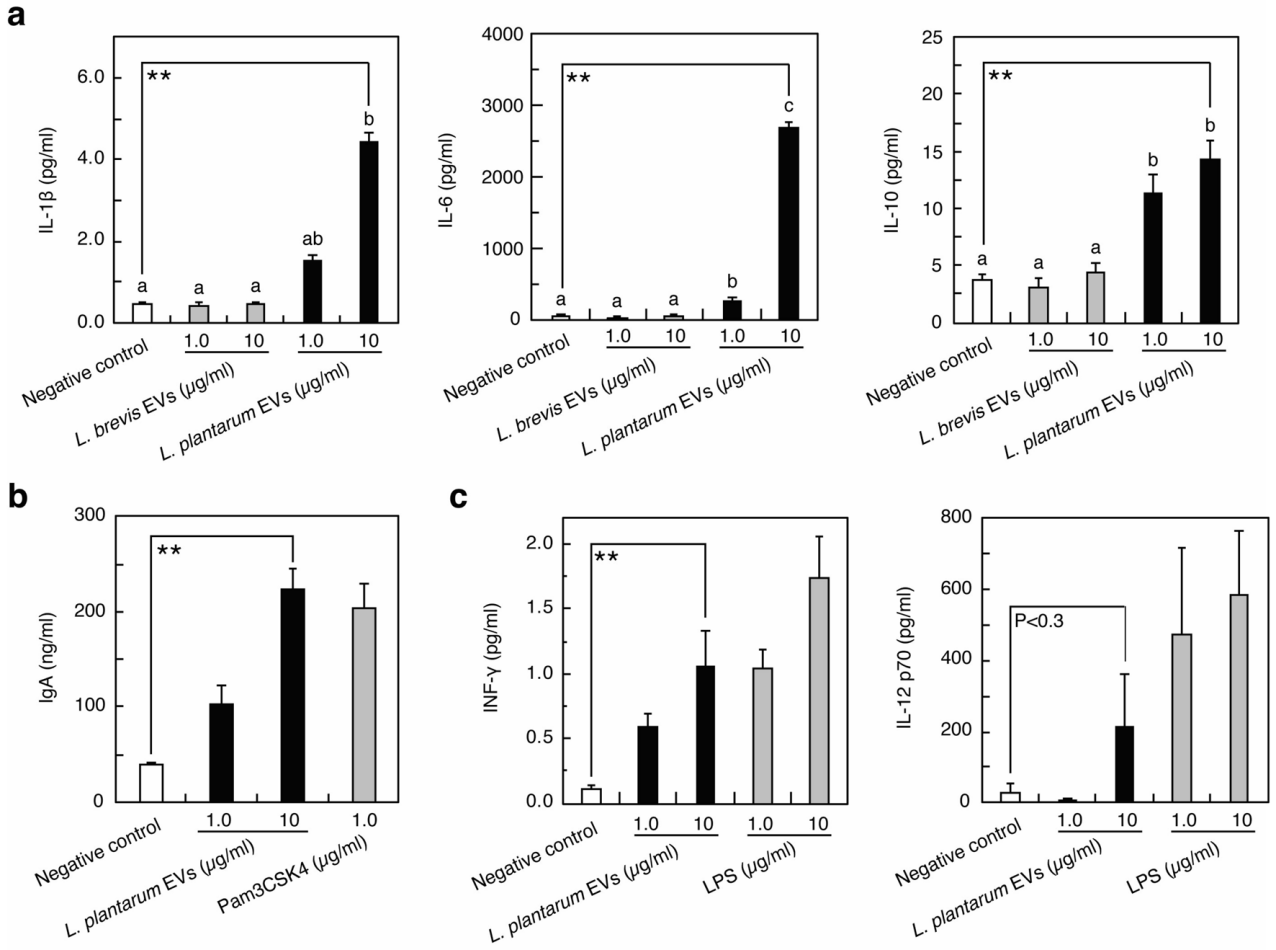
Stimulation of immune cells by *L*. *plantarum* EVs. The production of cytokines from Raw264 cells (**a,c**) and IgA from PP cells (**b**) was indicated. Pam3CSK4 (**b**) and LPS (**c**) were used as positive controls. PBS (**a–c**) was used as the negative control. Means ± SD, n = 3. (**a**) Bars identified by the same letters are not significantly different from each other (P > 0.01) by the Tukey’s test. (**a–c**) the Dunnett’s test, **P < 0.01 compared with negative control.

The effects of lactobacilli via EVs on host immune cells remain unclear. We investigated the response of immunocompetent cells elicited by *L*. *plantarum* EVs. Representative *L*. *plantarum* EVs were prepared from the supernatants of three independent cultures. As shown in Fig. 5a, the production of the pro-inflammatory cytokines IL-1β and IL-6 and the anti-inflammatory cytokine IL-10 from Raw264 cells was induced by *L*. *plantarum* EVs. When innate immunity is stimulated, immune cells produce cytokines. The results shown in Fig. 5a confirmed that innate immunity was stimulated by *L*. *plantarum* EVs. The amount of each cytokine induced by *L*. *plantarum* EVs was higher than that induced by *L*. *brevis* EVs. As shown in Fig. 5b, PP cells containing macrophages and B cells produced IgA in the presence of *L*. *plantarum* EVs, indicating that acquired immunity was stimulated by the addition of EVs. *L. sakei* EVs stimulated dendritic cells to produce IL-6, nitric oxide, and retinoic acid, which play important roles in enhancing IgA production by PP cells^8,9^. Since *L*. *plantarum* EVs induced the production of IL-6 from Raw264 cells, IL-6 appeared to be involved in the production of IgA from PP cells, similar to *L*. *sakei* EVs. To summarize, *L*. *plantarum* EVs induced the production of IFN-γ and IL-12 (Fig. 5c), which are involved in the differentiation of naive T-helper cells into T-helper type 1 cells^25^, suggesting that *L*. *plantarum* EVs activate both innate and acquired immune responses.

### Identification of cellular receptors that recognize *L*. *plantarum* EVs and their ligands

**Figure 6:**
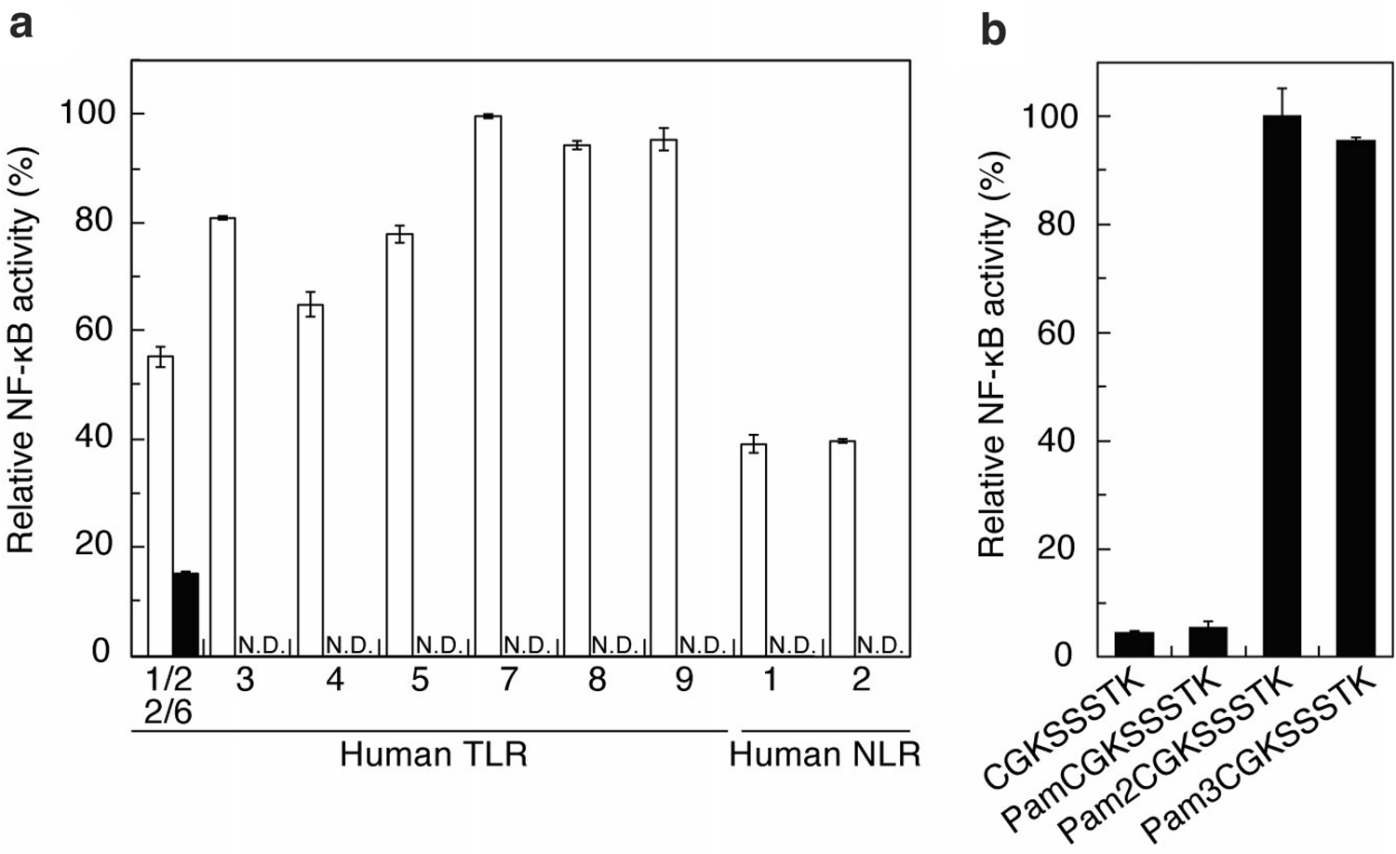
Human cell receptors involved in the recognition of *L*. *plantarum* EVs and lipopeptides from Lp19180. The recognition abilities of (**a**) *L*. *plantarum* EVs (closed bar) and each positive control (open bar, described in the Materials and Methods) and (**b**) acylated *N*-terminal peptides derived from Lp19180 were evaluated based on NF-κB-inducible SEAP activity in duplicate wells. Means ± SD. *N.D.* not detected.

*L*. *plantarum* produces EVs that stimulate host immune cells. The host cell receptors that recognize the EVs of lactobacilli and their ligands have remained unknown. Bacterial components are recognized by the PRRs of host cells. Therefore, we focused on PRRs to identify the host receptor for *L*. *plantarum* EVs. In the signaling pathway of immune cells, the recognition of ligands by PRRs has been shown to activate the transcription factor NF-κB^26^. Activated NF-κB then induces the expression of various cytokines downstream of the signaling pathway. Therefore, we examined whether *L*. *plantarum* EVs activate NF-κB via the recognition of PRRs, namely, human TLRs and NLRs. To assess the activation of NF-κB via TLRs and NLRs, human embryonic kidney (HEK293) cells expressing NF-κB-inducible secreted extracellular alkaline phosphatase (SEAP) and each of the human TLRs (TLR2, 3, 4, 5, 7, 8, or 9) and human NLRs (NLR 1 or 2) were used. As shown in Fig. 6a, *L.* *plantarum* EVs were recognized by TLR1/2 or TLR2/6 on the HEK293 cell surface (Fig. 6a, closed bar) and not by TLR4 or 5 on the HEK293 cell surface or by TLR3, 7, 8, or 9 or NLR1 and 2 inside HEK293 cells. TLR1, 2, and 6 have been shown to form heterodimers (TLR1/2 and TLR2/6) on the HEK293 cell surface^26^. Furthermore, previous studies demonstrated that *L*. *sakei* EVs and *L*. *rhamnosus* JB-1 EVs stimulated macrophages and dendritic cells, respectively, via TLR2 recognition^9,23^. Therefore, TLR1/2 and/or TLR2/6 appear to play an important role in the activation of host immune cells by lactobacilli EVs.

*S*. *aureus* Newman produces EVs containing small RNAs and these EVs are delivered to macrophages and trigger IFN-β responses via TLRs 3, 7, and 8, which recognize exogenous RNA^27^. As shown in Fig. 4, *L*. *plantarum* EVs contained small RNAs derived from rRNA, mRNA, tRNA, and other non-coding RNA similarly as found in *S*. *aureus* Newman EVs. However, we did not detect the activation of NF-κB via TLR3, 7, and 8 by *L*. *plantarum* EVs (Fig. 6a). Since the ligands recognized by host cell receptors differed among each EV, the ligands in each EV and the receptors in host cells need to be identified for the elucidation of host responses.

The EVs of *S*. *aureus* strain 6571 and *L*. *rhamnosus* JB-1 contain PGN and LTA, respectively, which are derived from the respective bacterial cell walls^22,23^. PGA and LTA in these EVs are presumably involved in the production of pro-inflammatory cytokines via TLR2. On the other hand, cell wall-derived LTA and PGN components were not detected in *L*. *plantarum* EVs (Table 1 and Supplementary Table S1). NF-κB was not activated via NLR1 or 2, which recognize iE-DAP and MDP, respectively, derived from PGN. In addition to LTA and PGN, LPs are ligands for TLR2^22,28^. We identified Lp19180 (GenBank accession No. LC633877) as a novel Lp in *L*. *plantarum* EVs (LOCUS_19180, Supplementary Table S3). In the proteomic analysis described above, Lp19180 of *L*. *plantarum* was assigned as a D-methionine transport system substrate-binding protein (MetQ) involved in the uptake of D-methionine^29^. In *L*. *plantarum* EVs, Lp19180 has been suggested to function as the TLR2 ligand. The nucleotide and estimated amino acid sequence of the Lp19180 gene are shown in Fig. S1 of Supplementary Material. Bacterial Lp generally localizes to the cytoplasmic membrane by removing the signal peptide and acylating the *N*-terminal cysteine with fatty acids^11^. Di- and tri-acylated Lps are located on the bacterial cell surface. In the case of Lp19180, the peptides detected in *L*. *plantarum* EVs using LC–MS/MS are shown in Fig. S1 (underlined) of Supplementary Material. We presumed that Cys22 was acylated and the polypeptide from Cys22 to Asp274 was presented outside the membrane of EVs towards the extracellular environment. As shown in Fig. 6b, using acylated *N*-terminal peptides derived from Lp19180, the TLR2-mediated activation of NF-κB was evaluated with HEK293 cells expressing NF-κB-inducible SEAP and TLR2. The results obtained showed that NF-κB was activated via TLR2 recognition by the diacylated *N*-terminal peptide (Pam2CGKSSSTK) and triacylated *N*-terminal peptide (Pam3CGKSSSTK). NF-κB was hardly activated by the non-acylated *N*-terminal peptide (CGKSSSTK) or monoacylated *N*-terminal peptide (PamCGKSSSTK). These results revealed that the di- and tri-acylated *N*-terminal peptide moieties of Lp19180 functioned as ligands for TLR2. Although the activation of NF-κB via TLR2 has been reported using the culture supernatants of *Lactobacillus paragasseri* K7, *Limosilactobacillus fermentum* L930BB, *Bifidobacterium animalis* IM386, and *L*. *plantarum* WCFS1, the ligands for NF-κB activation remain unclear^30^. In the present study, we detected EVs as novel functional particles in the culture supernatant of *L*. *plantarum*, demonstrated that *L*. *plantarum* EVs activated NF-κB via TLR2, and identified lipopeptides derived from Lp19180 as novel TLR2 ligands in *L*. *plantarum* EVs.

### Detection of EVs in the rat gastrointestinal tract

We demonstrated that immune cells were stimulated by the lipopeptide moiety of Lp19180 in *L*. *plantarum* EVs. Although previous studies reported that EVs from lactobacilli were taken up by M cells into the lamina propria^8,31^, the particle numbers and sizes of EVs in the gastrointestinal tract have not yet been directly measured. Therefore, we herein attempted to evaluate the particle numbers and sizes of EVs prepared from the gastrointestinal tract of Sprague–Dawley (SD) rats. As shown in Fig. S2 of Supplementary Material, the intestinal content (1 g) of the gastrointestinal tract contained 1.2 × 10^13^ particles of EVs with a mean particle size of 160 nm and a mode particle size of 132 nm. Feces from mice and humans contain EVs with mean particle sizes of 118 and 120 nm, respectively^32^. The particle sizes of EVs in the rat gastrointestinal tract are similar to those of fecal EVs from mice and humans. We demonstrated that extremely large amounts of EVs were present in the rat gastrointestinal tract. The intraperitoneal administration of mouse fecal EVs to mice has been shown to induce TLR2- and TLR4- mediated inflammation in local and systemic immunities^32^. Similarly, large amounts of EVs, including lactobacillus EVs, in the mouse gastrointestinal tract may be transferred to the lamina propria and affect the host immune system.

## Discussion

To clarify the immune cell responses induced by enterobacterial EVs, host cell receptors to EVs, and ligands present in EVs, we herein examined the biophysical parameters of *L*. *plantarum* EVs and comprehensively identified the biochemical components of EVs. The production of TNF-α from peritoneal exudate cells was previously shown to be induced by whole cells of *L*. *plantarum* JCM8341^13^. In the present study, we demonstrated that EVs produced by *L*. *plantarum* JCM8341 in culture medium induced the production of diverse pro- and anti-inflammatory cytokines. Host immune cell responses induced by *L*. *plantarum* EVs appeared to differ from those induced by whole *L*. *plantarum* cells. Therefore, it is necessary to characterize not only whole bacterial cells, but also EVs produced during the bacterial life cycle.

We herein reported the presence of a large amount of EVs in the rat intestinal tract for the first time. Although EVs in the rat intestinal tract appear to include EVs from enterobacteria, rat cells, and diets, the classification of EVs in the intestinal tract has not yet been performed. Further studies are needed to investigate the effects of bacterial EVs in the intestinal tract on the host organism. Some lactobacilli EVs may be taken up by M cells into the intestinal lamina propria^8,31^ and stimulate host immune cells via TLR2^9,23^. In host immune cells, TLR2 signal transduction may induce the production of pro-inflammatory cytokines by activating NF-κB through MyD88- and MAL/TIRAP-dependent pathways and anti-inflammatory cytokines through the PI3K/AKT pathway^26,33–36^. TLR2 polymorphisms have been shown to exert protective effects against intestinal mucosal damage^37,38^, while others may be associated with an increased risk of colorectal cancer^39,40^. Additionally, polymorphisms in human TLR2 have been strongly implicated in the development of several diseases, including rheumatoid arthritis, type I diabetes, and asthma^41,42^. Therefore, the stimulation of TLR2 leads to diverse physiological and cell reactions. Since lactobacilli EVs are recognized by TLR2, further research is needed to identify the molecules responsible for the TLR2 stimulation in EVs derived from gut microbes.

In *L*. *plantarum* EVs, we identified Lp19180 as a novel molecule that is recognized by TLR2. In whole cells of *L*. *plantarum*, Lp19180 has been suggested to play a role in the cellular uptake of D-methionine^29^. However, in *L*. *plantarum* EVs, the lipopeptide moiety of Lp19180 activated NF-κB via TLR2 recognition in host immune cells. FomA porin in *Fusobacterium nucleatum* EVs and cell wall muramidase in *L*. *casei* EVs were previously shown to activate NF-κB^43,44^, suggesting that EVs from various commensal bacteria in the gut environment contain a number of NF-κB activators. The activation of NF-κB plays a critical role in the maintenance of intestinal immune homeostasis^45–47^. Therefore, further studies are needed to identify TLR2-mediated NF-κB activators in EVs from the gut microbe.

In the present study, we propose the novel immunostimulatory effects of *L*. *plantarum*. As shown in Fig. 1, the TLR2-mediated activation of NF-κB by LP19180 in *L*. *plantarum* EVs elicited immunostimulatory effects, including innate and acquired immune cell responses. In future research, the immune cell responses induced by LP19180 and di- and tri- acylated *N*-terminal peptides need to be examined in more detail. Conclusively, lactobacilli EVs containing functional molecules appear to contribute to the maintenance of host intestinal immune homeostasis in addition to whole cells of lactobacilli. The elucidation of host cell responses induced by enterobacterial EVs and the identification of TLR ligands in enterobacterial EVs will enhance our understanding of the effects of symbiotic bacteria on host cells in the gut environment. Furthermore, the characterization of EVs will contribute to the development of new functional molecules for the regulation of physiological reactions.

## Materials and methods

### Bacterial culture and EVs preparation

*L*. *plantarum* JCM8341 was cultured in de Man, Rogosa, and Sharpe medium (Difco Laboratories) at 30 °C for 4 days under static culture conditions. *L*. *plantarum* EVs were then isolated from the culture (1000 ml) according to previously reported guidelines^48^. After centrifugation at 6000×*g* at 4 °C for 30 min to remove bacterial cells, the culture supernatant was centrifuged at 10,000×*g* at 4 °C for 30 min. The culture supernatant was then filtrated through 0.2-µm polyethersulfone filters (Sartorius, Göttingen, Germany) to remove cellular debris. The EVs of the filtered supernatant were concentrated by an Amicon Ultra Centrifugal filter device (cut-off: 100 kDa). The concentrated cell-free supernatant containing EVs was ultracentrifuged at 100,000×*g* at 4 °C for 2 h with Himac CP-80β (HITACHI, Tokyo, Japan). EVs were recovered as precipitates by ultracentrifugation and resuspended in Tris-buffered saline (TBS, pH 7.4, Nacalai Tesque, Kyoto, Japan). The EVs of *L*. *rhamnosus* JCM2772, *L casei* JCM 1134, and *L*. *brevis* JCM1059^T^ were prepared in the same manner. EVs were quantified by staining with the dye FM4–64 (5 µg/ml, Molecular Probes, Chicago, IL) at room temperature for 20 min. The fluorescence intensity of FM4-64 in the membranes of EVs was measured by an RF-6000 fluorescence spectrophotometer (Shimadzu, Kyoto, Japan) with an excitation wavelength of 515 nm and an emission wavelength of 635 nm. DNA, RNA, and protein concentrations in EVs were quantified using a Qubit 3.0 fluorometer (Life Technologies, Grand Island, NY).

### Physicochemical analysis of *L*. *plantarum* EVs

EVs were labeled with FM4-64 and the enumeration and sizing of labeled and non-labeled EVs were performed with a ViewSizer 3000 (software version 1.9.0.4518, HORIBA, Kyoto, Japan) using a 520-nm laser and a 650-nm filter. The zeta potential of EVs was assessed by a zeta potential analyzer (ELSZ-2000, Otsuka Electronics, Osaka, Japan) at 25 °C. EVs suspended in TBS (pH 7.4, Nacalai Tesque) were deposited on a carbon film-coated copper grid treated by glow discharge and then stained with 2% uranyl acetate solution for negative staining. The prepared grid was observed with the transmission electron microscope JEM-1400 (JEOL Inc., Tokyo, Japan).

### Metabolomic analysis of *L*. *plantarum* EVs

*L*. *plantarum* EVs (50 mg wet weight) were washed three times with 10 ml of TBS by ultracentrifugation and then disrupted with 75% MeOH and zirconia beads. The homogenate was centrifuged at 20,000×*g* for 10 min and the supernatant obtained was loaded on a MonoSpin C18 column (GL science, Tokyo, Japan) and eluted with 75% MeOH. The eluted fraction was analyzed using a HPLC Ultimate 3000 RSLC (Thermo Fisher Scientific, Waltham, MA, USA) coupled with a Q Exactive high-resolution mass spectrometer (Thermo Fisher Scientific). The scan range of mass spectrometry was set at *m*/*z* 80–1200. The detection and alignment of the peak were performed using ProteoWizard (https://proteowizard.sourceforge.io) and PowerGetBatch software^49^. The UC2 database (http://webs2.kazusa.or.jp/mfsearcher/uc2/) was used to identify metabolites.

### Genome analysis of *L*. *plantarum*

The genome of *L*. *plantarum* JCM8341 was sequenced using the Illumina HiSeq sequencer. All assembly genome sequence data and 16S rRNA sequence data were deposited in the NCBI GenBank with accession No. BPFY01000000 and LC633333, respectively. Gene predictions and functional annotation were conducted by the DDBJ Fast Annotation Pipeline (D-fast, https://dfast.ddbj.nig.ac.jp).

### Proteomic analysis of *L*. *plantarum* EVs

*L*. *plantarum* EVs (2.0 µg) were reduced with DTT and alkylated with iodoacetamide. Alkylated EVs were then digested with trypsin. The tryptic peptide mixture was desalted and applied to an Orbitrap Q Exactive Plus mass spectrometer through an EASY-nLC 1200 System (Thermo Fisher Scientific). To identify proteins, all MS/MS spectra obtained by the ESI mass analysis in the positive ion mode were analyzed using the MASCOT server (Matrix Science). Predicted protein sequence data sets were based on the annotation of the genome sequence of *L*. *plantarum* JCM8341, as described above. Protein identification was performed with scaffold software (version 5.0.0, Proteome Software, Inc., Portland, OR).

### Transcriptomic analysis of *L*. *plantarum* EVs

To evaluate the protection of RNA, the amount of RNA in RNase-treated EVs was compared with that of untreated EVs. *L*. *plantarum* EVs containing 1390 µg of RNA were treated with RNase I_f_ (250 U, NEB, Beverly, MA). Treated EVs was then ultracentrifuged at 100,000×*g* at 4 °C for 2 h with Himac CP-80β (HITACHI, Tokyo, Japan) and the pellet was resuspended in 400 µl of TBS (pH 7.4). Using RNase-treated EVs and untreated EVs, the quantification of RNAs and EVs was performed with a Qubit RNA assay kit and FM4-64, respectively. Small RNA-seq was performed to elucidate nucleotide sequences and measure RNA lengths. Total RNA was purified from *L*. *plantarum* EVs using an RNeasy plus universal mini kit (Qiagen, Hilden, Germany) and quantified using a NanoDrop spectrophotometer (Nanodrop, Wilmington, NC). RNA integrities (RIN; 2.6) were assessed and small RNAs were detected using an Agilent 2100 Bioanalyzer (Agilent Technologies, Santa Clara, CA). RNA-seq was performed using HiSeq (Illumina) with the library constructed by a TruSeq small RNA Library Prep Kit (Illumina). The 406,312 reads obtained were mapped to the reference genome sequence of *L*. *plantarum* using Bowtie software (version 1.3.1). Read abundance mapping to the reference genome sequence was normalized by the RPM calculation. All RNA seq data were deposited in the NCBI GenBank with accession No. DRX285247.

### Quantitative assessment of cytokines and IgA

In a quantitative assessment of cytokines, RAW264 cells (RCB0535, Riken Bioresource Center Cell Bank, Ibaraki, Japan) were cultured in Dulbecco’s Modified Eagle’s Medium (D-MEM; Wako Pure Chemical Industries, Ltd., Tokyo, Japan) with 10% fetal bovine serum (Thermo Fisher Scientific, Waltham, MA), 100 U/ml penicillin–streptomycin (Thermo Fisher Scientific), and 2 mM L-glutamine for 3 days. RAW264 cells were then seeded at 5.0 × 10^5^ cells/well on 96-well plates with or without EVs and incubated at 37˚C for 1 day under 5% CO_2_ in air. After the incubation, the culture supernatant was collected, and the concentrations of cytokines were measured using a V-PLEX Proinflammatory Panel 1 Mouse Kit (Meso Scale Discovery, Gaithersburg, MD). LPS from *Escherichia coli* O111:B4 (Sigma-Aldrich, St. Louis, MO) was used as the positive control. To prepare PP cells from female BALB/c mice, animal experiments were approved by the Animal Ethics Committee of Kansai University (Approval No. 1715) and conducted in compliance with the ARRIVE guidelines (http://www.nc3rs.org.uk/page.asp?id=1357). The preparation of PP cells and quantitative assessment of IgA were performed as previously reported^9^. PP cells (1.0 × 10^5^ cells/well) were inoculated into 96-well plates (Thermo Fisher Scientific) with or without EVs at 37˚C for 4 d under 5% CO_2_ in air. After the incubation, the culture supernatant was collected. IgA concentrations in the culture supernatant were measured by ELISA using 96-well plates (MaxiSorp; Thermo Fisher Scientific) coated with 10 µg/ml goat anti-mouse IgA (Bethyl Laboratories, Montgomery, TX). Purified mouse IgA (κ isotype control; BD) was used as the standard. Pam3CSK4 (InvivoGen, San Diego, CA) was used as the positive control.

### Recognition of *L*. *plantarum* EVs and lipopeptides by PRRs

Ligand screening was performed using HEK293 cell lines expressing NF-κB-inducible SEAP and each of the TLRs (HEK-Blue™ TLR2, 3, 4, 5, 7, 8, and 9) and NLRs (HEK-Blue™ NLR1 and 2). Recombinant HEK293 cells (5 × 10^5^ cells/well) were plated on 96-well plates and EVs (50 µg/ml) were then added and incubated at 37 °C for 18 h. The evaluation of EVs as PRR ligands was performed by measuring induced SEAP activity, which was quantitated spectrophotometrically using a SEAP reporter assay kit (HEK-Blue™ Detection, InvivoGen). The following ligands were used as positive controls; Pam2CysK4 (1 ng/ml) for TLR2, poly I:C (1 µg/ml) for TLR3, LPS-EK (10 ng/ml) for TLR4, flagellin (1 µg/ml) for TLR5, R848 (1 µg/ml) for TLR7, TL8-506 (1 µg/ml) for TLR8, ODN 2006 (10 µg/ml) for TLR9, C12-iE-DAP (10 µg/ml) for NLR1, and MDP (1 µg/ml) for NLR2 (InvivoGen). *N*-terminal peptides (1.3 µg/ml) derived from Lp19180 as PRR ligands were evaluated using the synthetic peptide cysteinyl-glycinyl-lysyl-serinyl-serinyl-serinyl-threoninyl-lysine (CGKSSSTK, Eurofins Genomics, Tokyo, Japan), *N*-palmitoyl-cysteinyl-glycinyl-lysyl-serinyl-serinyl-serinyl-threoninyl-lysine (PamCGKSSSTK, Eurofins Genomics), *S*-[2,3-di(palmitoyl)-propyl]-cysteinyl-glycinyl-lysyl-serinyl-serinyl-serinyl-threoninyl-lysine (Pam2CGKSSSTK, Eurofins Genomics), and *N*-palmitoyl-*S*-[2,3-di(palmitoyl)-propyl]-cysteinyl-glycinyl-lysyl-serinyl-serinyl-serinyl-threoninyl-lysine (Pam3CGKSSSTK, Eurofins Genomics).

### Statistics

The resulting values were expressed as mean ± standard deviation (SD). Statistical analysis was performed using one-way ANOVA followed by the Dunnett’s test compared to the negative control and the Tukey’s test. P-value < 0.01 was considered statistically significant based on the Dunnett’s test and the Tukey’s test.

### Statement

All methods were carried out in accordance with relevant guidelines and regulations.

## Supplementary Information

Supplementary Information 1.

Supplementary Information 2.

Supplementary Table S1.

Supplementary Table S2.

Supplementary Table S3.

Supplementary Table S4.
