# Supplementary material for: Characterization of extracellular vesicles from Lactiplantibacillus plantarum
Source: Sci Rep. 2022 Aug 8;12:13330. doi: 10.1038/s41598-022-17629-7 (PMC9360025; doi:10.1038/s41598-022-17629-7)
Supplement: Supplementary file 1 — Supplementary Information 1. [file 41598_2022_17629_MOESM1_ESM.pdf]

① The four wells for the samples could be confirmed.

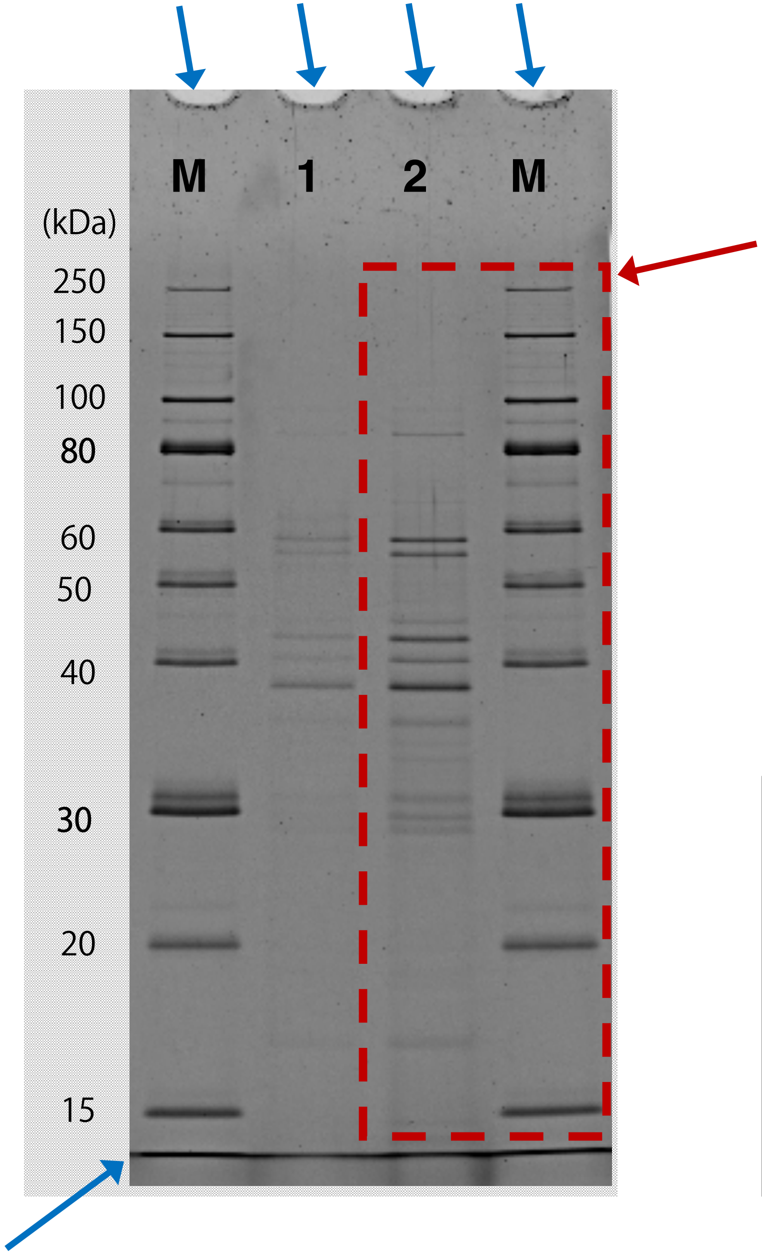

③ The area shown by the red dotted line is used in the Fig. 3a. This original gel image was flipped left and right and used in Fig. 3a.

M: Marker

Lane 1: *L. plantarum* EVs (0.3 μg) were quantified by qubit protein assay kit (Life Technologies).

Lane 2: *L. plantarum* EVs (0.3 μg) were quantified by BCA protein assay kit (Thermo Fisher Scientific) .

② The line was observed at the tip of the gel after electrophoresis.
