# Supplementary material for: Characterization of extracellular vesicles from Lactiplantibacillus plantarum
Source: Sci Rep. 2022 Aug 8;12:13330. doi: 10.1038/s41598-022-17629-7 (PMC9360025; doi:10.1038/s41598-022-17629-7)
Supplement: Supplementary file 2 — Supplementary Information 2. [file 41598_2022_17629_MOESM2_ESM.docx]

**Characterization of extracellular vesicles from *Lactiplantibacillus plantarum***

Atsushi Kurata^1*^, Shogo Kiyohara^1^, Tomoya Imai^2^, Shino Yamasaki-Yashiki^3^, Nobuhiro Zaima^1,4^, Tatsuya Moriyama^1,4^, Noriaki Kishimoto^1^, and Koichi Uegaki^1,4^

^1^Department of Applied Biological Chemistry, Faculty of Agriculture, Kindai University, 204-3327 Nakamachi, Nara, Nara 631-8505, Japan.

^2^Research Institute for Sustainable Humanosphere, Kyoto University, Uji, Kyoto 611-0011, Japan.

^3^Department of Life Science and Biotechnology, Faculty of Chemistry, Materials and Bioengineering, Kansai University, 3-3-35 Yamate-cho, Suita, Osaka 564-8680, Japan.

^4^Agricultural Technology and Innovation Research Institute, Kindai University, 204-3327 Nakamachi, Nara, Nara 631-8505, Japan.

*Corresponding author e-mail address: kurata090401@nara.kindai.ac.jp

**Supplementary Information**

**Supplementary Materials and Methods**

1. Amino acid and nucleotide sequences of Lp19180

The Lp19180 gene product (GenBank accession No. LC633877) consisted of two parts, signal peptide domain (Met1-Gly21) and substrate-binding domain (Cys22-Asp274), according to predictions from a conserved domain search (http://www.ncbi.nlm. nih.gov/Structure/cdd/wrpsb.cgi). The trypsin-digested peptide sequences identified by LC-MS/MS are underlined. A signal peptide domain may be cleaved between Gly21 and Cys22 (predicted by the database of SignalP V5.0, https://services.healthtech.dtu.dk/service.php?SignalP-5.0) to acylated at Cys22 for localization to the cell membrane of *L*. *plantarum*.

2. Preparation of EVs in rat intestinal contents

The animal experiment was approved by the Kindai University Animal Care and Use Committee (Approval No. KAAG-25-001). Six-week-old female SD rats (Japan SLC, Inc., Shizuoka, Japan, n=3) were maintained in a room at 25±1℃ with a 12-h light/dark cycle with free access to a normal diet (0.25% choline chloride, 0.3% cysteine, 1% AIN-93 vitamin mix, 3.5% AIN-93G mineral mix, 5% cellulose, 10% sucrose, 20% casein, 55.75% cornstarch, and 4.2% coconut oil) and water. SD rats were kept under general dietary conditions. After 5 weeks, rats were euthanized by an overdose of pentobarbital sodium and the intestines were collected. The rat intestinal content was suspended in TBS (pH7.4, Nacalai Tesque) at a concentration of 1 g/ml. The preparation of EVs using the suspension was performed in the same manner as that using the culture medium of *L*. *plantarum*. EVs were collected from the supernatants of three independent rat intestinal contents. EVs were diluted 1:1000 in TBS (pH7.4, Nacalai Tesque). The enumeration and sizing of EVs were then performed at 25˚C with a Nanosight NS300 system (Malvern Instruments Ltd., Worcestershire, UK) using software NTA 2.3 (n=3).

**Supplementary Figures**


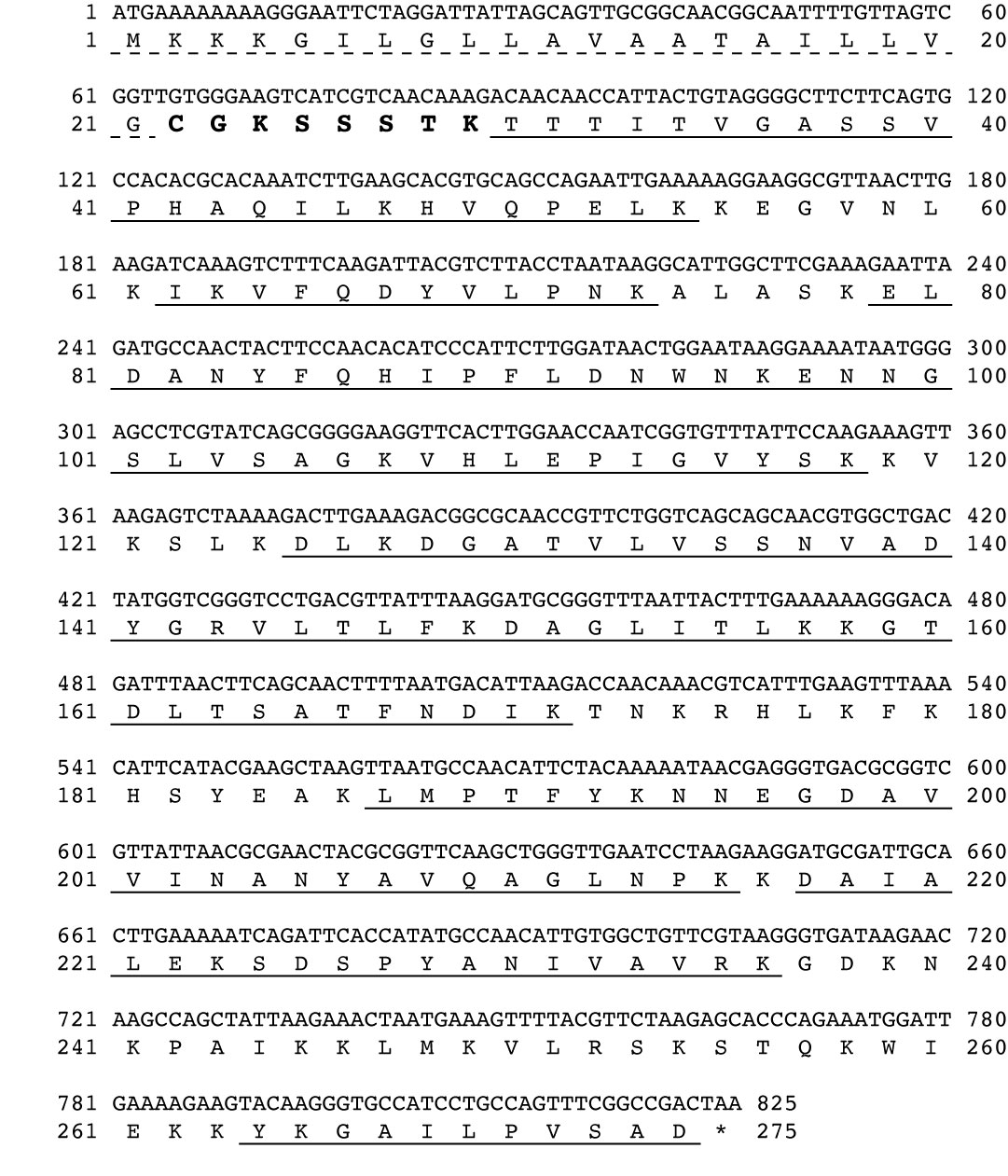


**Fig. S1.** Amino acid and nucleotide sequences of Lp19180

The signal peptide (dashed line) and *N*-terminal peptide (bold) of mature Lp19180 are indicated. The underlined peptides (Thr30 - Lys54, Ile62 - Lys73, Glu79 - Lys118, Asp125 - Lys171, Leu187 - Lys215, Asp217 - Lys236, and Tyr264 - Asp274) were detected by LC-MS/MS. By using SignalP (https://services.healthtech.dtu.dk/service.php?SignalP-5.0), the peptide (Met1-Gly21) may be predicted as the signal peptide.


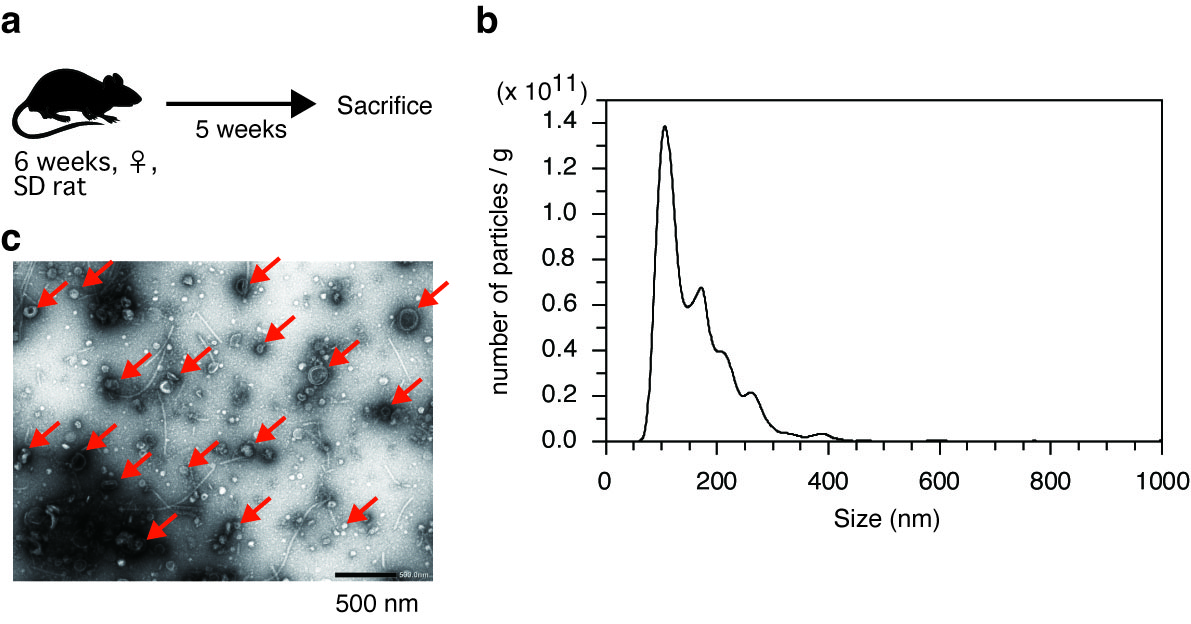


**Fig. S2.** EVs in the intestinal tracts of rats

(**a**) Schema of the experiment. (**b**) Size distributions of EVs detected in the intestinal tract of SD rats. Means (n=3) were indicated. (**c**) TEM images of EVs that are detected in the intestinal tracts of SD rats. Red arrows indicate EVs.
